# A bismuth triiodide monosheet on Bi_2_Se_3_(0001)

**DOI:** 10.1038/s41598-019-40506-9

**Published:** 2019-03-11

**Authors:** Andrey Polyakov, Katayoon Mohseni, German R. Castro, Juan Rubio-Zuazo, Alexander Zeugner, Anna Isaeva, Ying-Jiun Chen, Christian Tusche, Holger L. Meyerheim

**Affiliations:** 1 Max-Planck-Institut für Mikrostukturphysik, Weinberg 2, 06120 Halle, Germany; 2 SpLine, Spanish CRG BM25 Beamline at the ESRF (The European Synchrotron), F-38000 Grenoble, France; 3 Instituto de Ciencia de Materiales de Madrid, Consejo Superior de Investigaciones Científicas (ICMM-CSIC), 28049 Madrid, Spain; 4 Department of Chemistry and Food Chemistry, TU Dresden, Helmholtzstraße 10, 01069 Dresden, Germany; 5 Technische Universität Dresden, Institut für Festkörper- und Materialphysik, Helmholtzstraße 10, 01069 Dresden, Germany; 6 Leibniz-Institut für Festkörper-und Werkstoffforschung Dresden, Helmholtzstraße 20, 01069 Dresden, Germany; 7 Forschungszentrum Jülich GmbH, Peter Grünberg Institut (PGI-6), 52425 Jülich, Germany; 8 Fakultät für Physik, Universität Duisburg-Essen, 47057 Duisburg, Germany

## Abstract

A stable BiI_3_ monosheet has been grown for the first time on the (0001) surface of the topological insulator Bi_2_Se_3_ as confirmed by scanning tunnelling microscopy, surface X-ray diffraction, and X-ray photoemision spectroscopy. BiI_3_ is deposited by molecular beam epitaxy from the crystalline BiTeI precursor that undergoes decomposition sublimation. The key fragment of the bulk BiI_3_ structure, ${{\rm{a}}}_{\infty }^{2}$[I—Bi—I] layer of edge-sharing BiI_6_ octahedra, is preserved in the ultra-thin film limit, but exhibits large atomic relaxations. The stacking sequence of the trilayers and alternations of the Bi—I distances in the monosheet are the same as in the bulk BiI_3_ structure. Momentum resolved photoemission spectroscopy indicates a direct band gap of 1.2 eV. The Dirac surface state is completely destroyed and a new flat band appears in the band gap of the BiI_3_ film that could be interpreted as an interface state.

## Introduction

Two-dimensional (2D) metal halides have been of interest since a long time for their intriguing and tunable properties. Bulk BiI_3_ has been studied intensely as a material for nuclear radiation detection applications and its photovoltaic properties^1,2^.

The periodic structure of BiI_3_ belongs to the inversion symmetric space group $R\bar{3}$ (no. 148) and consists of ${{\rm{a}}}_{{\rm{\infty }}}^{2}$[I—Bi—I] trilayers (TL’s) which are stacked in a rhombohedral *ABC* sequence^3^. Bulk BiI_3_ is an indirect band gap semiconductor with a gap width of 1.67 eV^1^ which is favorable for potential energy harvesting^4^. Owing to the presence of a high-Z element (Bi) spin-orbit coupling (SOC) is significant and new functionalities are expected by reducing the crystal’s dimensionality to the ultra-thin film limit which might involve a symmetry lowering, for instance by the loss of the inversion center induced by atomic relaxations.

Up to now, only theoretical studies on BiI_3_ monosheets have been carried out which have predicted its stability and the possibility to tune the band structure by strain^4,5^. However, no experimental investigation concerning structure and physical properties of BiI_3_ monosheets are available. One standard way to prepare a thin film of a layered material is cleaving the bulk crystal which in the case of BiI_3_ consists of weakly van-der-Waals (vdW) connected sheets. Its disadvantage is that this method neither allows precise control of the film thickness nor the preparation of a single monosheet. Furthermore, handling of bulk BiI_3_ is hindered due to its notable moisture-sensitivity.

Here we establish a different approach by depositing a monosheet of BiI_3_ on the (0001) surface of the topological insulator (TI) Bi_2_Se_3_ by using molecular beam epitaxy (MBE) from a Knudsen cell filled with presynthesized BiTeI single crystals heated to 380 °C. As a result, a stoichiometrically almost exact BiI_3_ film is deposited by decomposition sublimation of BiTeI, as corroborated by X-ray photoemission spectroscopy (XPS), scanning tunnelling microscopy (STM) and surface X-ray diffraction (SXRD). In addition, momentum resolved photoemission spectroscopy^6^ demonstrates the instability of the Dirac topological surface state (TSS) of the Bi_2_Se_3_ substrate upon BiI_3_ deposition and the appearance of flat band interface states in the direct band gap of BiI_3_.

## Results

### Deposition of BiI_3_ on Bi_2_Se_3_(0001)

Experiments were carried out *in-situ* in ultra-high-vacuum systems equipped with standard surface physics analytical tools.

**Figure 1:**
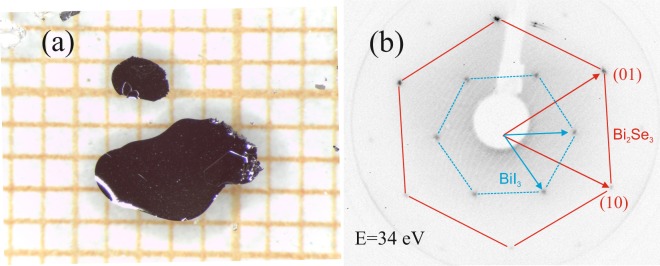
(**a**) CVT-grown single crystals of BiTeI. The squares are 1 × 1 mm^2^ in size. (**b**) LEED pattern for saturation coverage of BiI_3_ on Bi_2_Se_3_ collected at 34 eV kinetic energy. Hexagons indicate the first order spots of the Bi_2_Se_3_ substrate and BiI_3_, which approximately forms a √3 × √3 superstructure.

Figure 1(a) shows the BiTeI single crystal which was grown by chemical vapor transport (CVT) (see methods). BiI_3_ was MBE-deposited onto the substrate that was kept at room temperature. The underlying mechanism can be rationalized by decomposition sublimation of BiTeI^7–9^: $3\,{\rm{BiTeI}}({\rm{s}})\rightleftharpoons {{\rm{Bi}}}_{2}{{\rm{Te}}}_{3}({\rm{s}})+{{\rm{BiI}}}_{3}({\rm{g}})$ . It is well established that solid BiTeI congruently melts at 555 °C and its volatilization commences above approximately 300 °C primarily by an incongruent decomposition into solid Bi_2_Te_3_ and gaseous BiI_3_^7,8^. This is a typical behavior of chalcogenide halides^9^. The remaining Bi_2_Te_3_ may decompose further, forming subsidiary BiTe and Te_2_ gas species: $\frac{2}{5}\,{{\rm{Bi}}}_{2}{{\rm{Te}}}_{3}({\rm{s}})\rightleftharpoons \frac{4}{5}\,{\rm{BiTe}}({\rm{g}})+\frac{1}{5}\,{{\rm{Te}}}_{2}({\rm{g}})$.

Vapor transport of both BiTeI and Bi_2_Te_3_ can proceed as an alternative reaction with these species:

$$\text{BiTeI}({\rm{s}})\rightleftharpoons {\rm{BiI}}({\rm{g}})+\frac{1}{2}\,{{\rm{Te}}}_{2}({\rm{g}})$$

$${{\rm{BiTe}}}_{3}({\rm{s}})+{{\rm{BiI}}}_{3}({\rm{g}})\rightleftharpoons 3\,{\rm{BiI}}({\rm{g}})+\frac{3}{2}\,{{\rm{Te}}}_{2}({\rm{g}})$$

However, the partial pressures of BiI(g) and Te_2_(g) increase sufficiently only above 410 °C and can then promote efficient vapor transport^7^. At our working temperatures, the total pressure is largely dominated by BiI_3_ gas species for a broad range of compositions in the ternary Bi-Te-I phase diagram^10^.

Formation of a BiI_3_ film on the Bi_2_Se_3_ substrate was observed via appearance of new LEED spots that closely corresponded to a √3 × √3-R30° superstructure, as shown in Fig. 1(b), in which the substrate and the adlayer spots are highlighted by the large and small hexagon, respectively. Epitaxial growth of BiI_3_ on Bi_2_Se_3_ is feasible thanks to a close structural match between the bulk structures. Both crystal structures can be viewed as hexagonal close-packed arrangements of anions (either iodine or selenium) with cations (bismuth) occupying the octahedral voids in a regular fashion. In the tetradymite-type structure, like Bi_2_Se_3_, two consecutive rows of octahedral voids along the *c* axis are fully populated and each third row remains empty, thus forming a van der Waals (vdW) gap between the quintuple layers (QL), ${{\rm{a}}}_{{\rm{\infty }}}^{2}$[Se–Bi–Se–Bi–Se]. The latter are stacked in the *ABC*-sequence^11,12^. In BiI_3_, 2/3 of the octahedral sites within each second row of cations along the stacking direction are occupied. In the case of ideally matching lattices, BiI_3_ would exhibit a √3 × √3-R30° superstructure in the *ab*-plane with respect to the Bi_2_Se_3_ (0001) − (1 × 1) surface unit cell. But the existing difference in the van der Waals radii (Se: 1.82 Å vs. I: 2.04 Å) constitutes a mismatch between the anionic lattices, that becomes evident from the comparison of the respective unit cell parameters *a*: whereas *a*(Bi_2_Se_3_) = 4.134(2) Å^13^, *a*(BiI_3_) = 7.5249(3) Å^3^ is about 0.3 Å larger than *a*(Bi_2_Se_3_)√3. The detailed SXRD analysis of the deposited BiI_3_ indicates that the first order spot is located at *h* = *k* = 0.316 units of the hexagonal Bi_2_Se_3_ reciprocal surface lattice, rather than at $h=k=\frac{1}{3}$ for the ideal √3 × √3-R30° superstructure. Hence, the BiI_3_ film is incommensurate with the substrate just like in the bulk case. Moreover, the mismatch is even stronger in the film, since the adlayer has an in-plane lattice parameter of *a*_0_ = 7.56 Å as compared to the reported range of bulk values which lie between *a*_0_ = 7.5249 Å and 7.50 Å^3,14,15^. We note that the lateral strain is only +0.8% at most and is thus well below the computed breaking strain of 13% for a free standing monosheet^5^.

**Figure 2:**
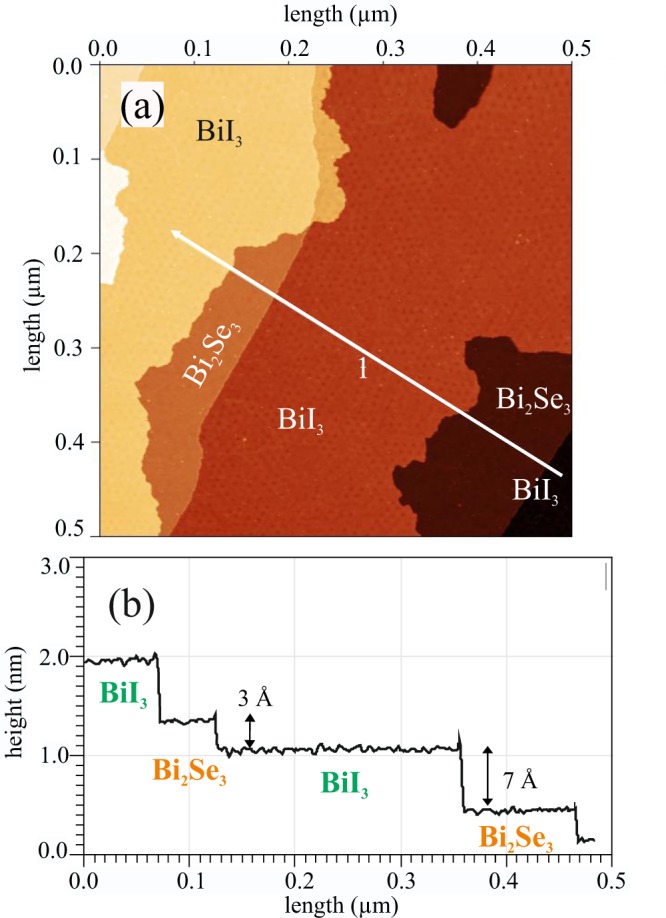
(**a**) 500 × 500 nm^2^ STM image (U = −2 V, I = 50 pA) of a single layer of BiI_3_ on Bi_2_Se_3_. (**b**) Profile along the white line labelled by “1” in (**a**).

Figure 2(a) shows a 500 × 500 nm^2^ STM image (U = −2 V, I = 50 pA) of a single-layer BiI_3_ film. The profile along the white line (labelled by “1”) is plotted in (b). The surface of the Bi_2_Se_3_ single crystal is characterized by 9.5 Å high steps which correspond to a full QL. The BiI_3_ monosheet has about 7 Å high steps corresponding to 1/3 of the bulk *c*_0_ lattice parameter of 20.72 Å related to three triple layers (TL)^3,14^. The wetting of the Bi_2_Se_3_ terraces proceeds from the step edges, leaving 3 Å high steps between the BiI_3_ layer and the next terrace.

### Surface X-ray diffraction

**Figure 3:**
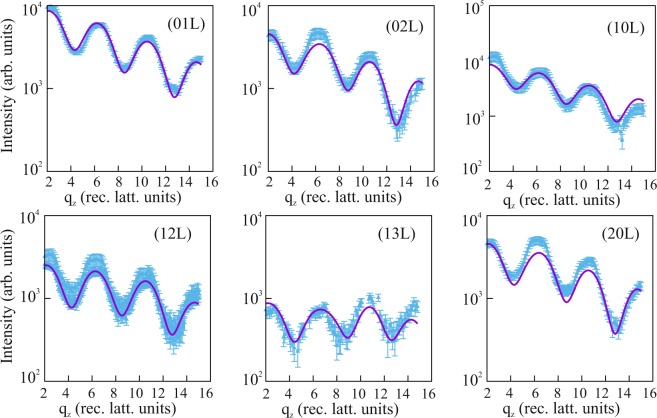
Experimental (symbols) and calculated (lines) reflection intensities on logarithmic scale along several reciprocal lattice rods of the BiI_3_ film.

The atomic structure of the deposited film was analyzed by SXRD experiments which were carried out at the beamline SpLine BM25b of the European Synchrotron Radiation Facility in Grenoble (France) using a UHV six-circle diffractometer equipped with a two-dimensional pixel detector. During BiI_3_ deposition, the first-order (h = k = 0.316) reflection intensity was monitored until saturation. Subsequently, reflection intensities along several lattice rods were collected under grazing incidence (*α*_*i*_ = 2°) of the incoming beam. In total 1042 symmetry independent reflections were collected along six lattice rods which are shown as symbols in Fig. 3. The intensities were fitted using the program “Prometheus”^16^ that enables refinement of anisotropic and anharmonic displacement parameters^17^ and crystal twinning. The latter is especially relevant in the present case owing to the two possible orientations of the adsorbate lattice relative to the substrate lattice, that forge the 6 *mm* point symmetry of the experimental diffraction pattern. In the plane group *p*3, bismuth atoms occupy the Wyckoff positions 1*a*, 1*b*, 1*c*, while iodine atoms reside in the general position 3*d*. Note that the plane group *p*3 lacks inversion symmetry in contrast to the space group $R\bar{3}$ of the bulk structure and in contrast to the $p\bar{3}$ plane group of individual BiI_3_ layers in the periodic structure^3^. The refined atomic coordinates and the isotropic displacement parameters (B = 8*π*^2^ <*u*>^2^) for the adlayer are listed in Table 1. The best fits to the experimental data according to these parameters are shown as solid lines in Fig. 3. The fit quality is quantified by the goodness of fit (GOF) and the un-weighted residuum which is given by: ${R}_{{\rm{U}}}=\sum |{I}_{{\rm{o}}{\rm{b}}{\rm{s}}}-{I}_{{\rm{c}}{\rm{a}}{\rm{l}}{\rm{c}}}|/\sum {I}_{{\rm{o}}{\rm{b}}{\rm{s}}}$. Here, *I*_obs_, *I*_calc_ are the experimental and calculated intensities, respectively and the summation runs over all datapoints. The GOF is given by: ${\rm{GOF}}=\sqrt{1/(N-P)\cdot \sum [({I}_{obs}-{I}_{calc}{)}^{2}/{\sigma }^{2}]}$, where the difference between observed and calculated intensities is normalized to the uncertainties expressed by the standard deviation (*σ*) and to (*N* − *P*), i.e. the difference between the number of *independent* data points (N) and the number of parameters (P) which are varied. Here, we derive GOF = 1.40 and R_*U*_ = 0.14, which are very reasonable values in view of the weak intensities scattered by the monosheet.

**Table 1:** Refined atomic coordinates, displacement parameters and occupancy factors (Θ) of the BiI_3_ film.

Atom type	Atom Nr.	x	y	z	B(Å^2^)	Θ
I	1	0.3344 (3)	0.3313 (4)	0.0000 (*)	1 (*)	1 (*)
Bi	2	0.0000 (*)	0.0000 (*)	0.0822 (5)	15 (1)	1 (*)
Bi	3	0.6666 (*)	0.3333 (*)	0.0769 (4)	22 (1)	1 (*)
I	4	0.3353 (4)	0.0029 (4)	0.1356 (5)	1 (*)	1 (*)
I	5	0.6727 (10)	0.0089 (10)	0.2783 (5)	1 (*)	1/2 (*)
Bi	6	0.0000 (*)	0.0000 (*)	0.3407 (4)	8 (1)	1/2 (*)
Bi	7	0.3333 (*)	0.6666 (*)	0.3256 (5)	4 (1)	1/2 (*)
I	8	0.6746 (13)	0.6710 (17)	0.4031 (5)	1 (0.1)	1/2 (*)

**Figure 4:**
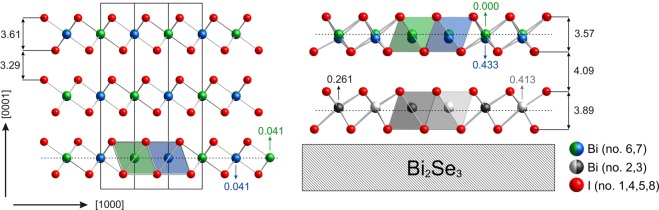
Crystal structure of bulk BiI_3_ (left) compared to the experimentally elucidated structure model of BiI_3_ film on Bi_2_Se_3_(0001) (right). The film consists of a complete first TL adjacent to the Bi_2_Se_3_ (0001) surface and a second TL, which is only half filled. Iodine atoms are depicted in red, the bismuth atoms are color-coded in accordance with their shifts from the central position within the octahedra (traced by a dotted line). Atoms are numbered according to Table 1. Interplanar distances and shifts of the bismuth atoms are given in Å. We estimate the uncertainty of the distance determination equal to 0.1 Å.

The atomic coordinates (x, y, z) in plane group *p*3 are given with respect to the unit cell parameters *a*_0_ = *b*_0_ = 7.56 Å, *c*_0_ = 28.65 Å. Note that c_0_ refers to the Bi_2_Se_3_ substrate. Standard deviations are given in brackets related to the last digit. Parameters labelled by an asterisk (*) are kept constant. Atoms are labelled according to Fig. 4.

Figure 4 shows a side view of the resultant structure model of the thin film in comparison with the periodic bulk structure of BiI_3_^3^. At first, the analysis shows that there is one complete TL and approximately one half of a second TL on the Bi_2_Se_3_ surface. Thus, in comparison with the STM experiments which indicated a single TL only, this sample is covered by BiI_3_ at its saturation coverage at 300 K. It can be concluded that the interlayer interaction is very weak which is also corroborated by the fact that desorption of the whole film sets in at about 360 K as evidenced by the rapid loss of the BiI_3_ reflection intensities. Correspondingly, the vertical spacing between the first and the half filled second TL is considerably enlarged as compared to the bulk. For instance, the closest interlayer I–I distance *across* the vdW gap increases from 4.14 Å in the bulk to 4.8 Å in the film.

The SXRD analysis also finds considerable relaxations within the TL’s. The first TL adjacent to the substrate is notably stretched as compared to its bulk counterpart, namely, the interplanar distance between the terminating iodine atoms (in other words, the height of a TL) increases from 3.61 Å (bulk) to 3.89 Å. The interatomic Bi–I distances towards the substrate (3.32–3.44 Å) are considerably longer than in the opposite direction (2.94–3.00 Å). All bismuth atoms (light and dark grey #2, 3 atoms in Fig. 4) are strongly displaced from the central position within the coordination octahedra towards the same side, namely, away from the substrate. The driving force of this movement cannot be pinpointed in a straightforward way, since the difference in electronegativity between iodine (2.66) and selenium (2.55) is not large, and both values are much higher than that for bismuth (2.02). One may tentatively assume that the I/Se interaction at the film/substrate interface -although weak- might lead to a weakening of the I–Bi bond and with it to an elongation of the corresponding bonds. Consideration of the van der Waals (Bi: 1.82 Å vs. I: 2.01 Å) and the (effective) ionic (Bi^3+^: 1.03 Å vs. I^−^: 1.70 Å) radii^18,19^ imposes limits of 3.86 Å and 2.73 Å for the Bi–I interatomic distance for atomic and ionic bonds, respectively. The distances derived from the SXRD analysis suggest a considerable ionicity of the Bi–I bond.

The second TL, which is only about half filled has a height of 3.57 Å which is much closer to the respective bulk value. The crystal structure of bulk BiI_3_ shows two distinct sets of Bi–I interatomic distances alternating in the first coordination polyhedron of each bismuth atom, i.e. 3.054 Å and 3.125 Å^3^. Such distortions ensure the highest density of TL packing^3^. Furthermore, the lone-pair effect on bismuth manifests itself in symmetrical shifts of adjacent Bi atoms in the opposite directions from the ideal site in the middle of the octahedral iodine environment (see Fig. 4). The same effects but with a larger magnitude are observed in the second TL. For instance, the Bi # 7 atom shifts by 0.4 Å from the middle position within an octahedron, and variations in the alternating longer and shorter interatomic Bi–I distances reach 0.5 Å. In general, the second TL of the BiI_3_ film demonstrates a bonding pattern characteristic for the bulk structure. One may argue that the formation of the bulk structure already commences as the second TL grows atop of the first TL, and that with it the accompanying bulk effects associated with the optimum packing emerge. The weak interlayer interaction nevertheless defines the mutual arrangement of the TL’s and their distortions, just like in the periodic crystal structure.

### Photoemission Spectroscopy

**Figure 5:**
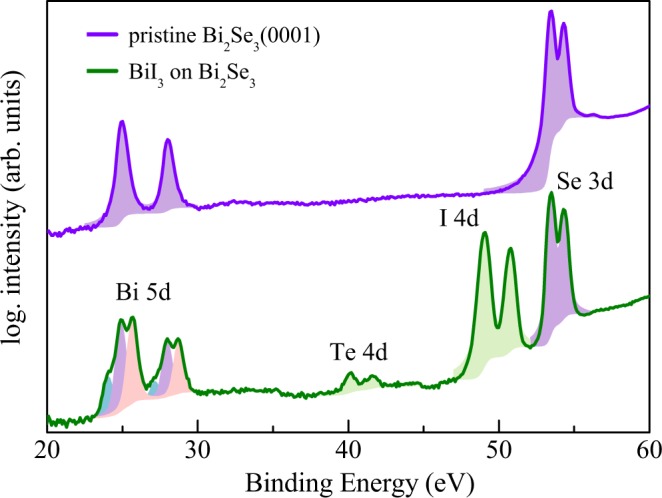
Comparison of X-ray photoemission spectra collected for pristine and BiI_3_-covered Bi_2_Se_3_ (0001) collected at a photon energy of h*ν* = 200 eV. The photoemission intensities are plotted on a log-scale and are shifted for clarity. Solid areas below the core level photoemission peaks indicate the fitted line shapes. Note the very low Te-4d emission indicating the presence of almost pure BiI_3_. The spin-orbit split Bi-5d lines exhibit a chemical shift of about 0.6 eV related to different Bi environments.

Owing to their very similar atomic number, X-ray diffraction is not capable to separate the atomic species iodine (Z = 53) and tellurium (Z = 52). X-ray photoemission spectroscopy was used to analyze the chemical composition of the film. All photoemission spectroscopy experiments were carried out at the NanoESCA beamline^20^ of the Elettra Synchrotron in Trieste (Italy). After film deposition at room temperature until saturation as in the case of the SXRD experiments the sample was kept at 130 K using s-polarized light and a photon energy of *hν* = 200 eV. Figure 5 compares two XPS spectra collected for the pristine and the BiI_3_ saturated Bi_2_Se_3_ (0001) surface. In the case of the pristine sample, the spin-orbit split Bi-5d (experimental binding energy E_*B*_ = 25.0 and 28.0 eV for 5d_5/2_ and 5d_3/2_, respectively) and the Se-3d (E_*B*_ = 53.5 and 54.3 eV for 3d_5/2_ and 3d_3/2_, respectively) are observed.

After film deposition in addition the 4d lines of iodine (E_*B*_ = 49.0 [4d_5/2_] and 50.8 eV [4d_3/2_]) and tellurium (E_*B*_ = 40.1 [4d_5/2_] and 41.6 eV [4d_3/2_]) appear. For a quantitative evaluation, the core level peaks were fitted using Voigt line shapes, taking into account the natural line width and the instrumental resolution, on top of a Shirley background. The fitted peak areas were subsequently corrected on the basis of tabulated photoionization cross sections and by a polarization factor for the photon energy of *hν* = 200 eV used for the XPS measurement^21^. This results in the normalized intensities of I(Se-3d_3/2_) = 1, I(I-4d_3/2_) = 2.2, I(Te-4d_3/2_) = 0.07, and I(Bi-5d_3/2_) = 2.3. Thus, the tellurium contribution in the film equals to only about 3% of that of the iodine contribution, indicating that the film is almost pure BiI_3_. In particular, the tellurium concentration is far away from that in stoichiometric BiTeI, for which the tellurium and iodine 4d signals are expected to be very similar.

Finally, the Bi-5d lines consist of totally three components, exhibiting a chemical shift due to a different bismuth environment in the film and in the substrate. The two most pronounced components are attributed to Bi in the Bi_2_Se_3_ substrate (${{\rm{E}}}_{B}^{3/2}$ = 28.0 eV and ${{\rm{E}}}_{B}^{\mathrm{5/2}}$ = 24.9 eV) and Bi atoms in the BiI_3_ film (${{\rm{E}}}_{B}^{\mathrm{3/2}}$ = 28.7 eV and ${{\rm{E}}}_{B}^{\mathrm{5/2}}$ = 25.7 eV). In addition, a third component can be identified at lower binding energy (${{\rm{E}}}_{B}^{\mathrm{3/2}}$ = 27.16 eV and ${{\rm{E}}}_{B}^{\mathrm{5/2}}$ = 24.1 eV), leading to a small shoulder of the Bi-5d line. The total contribution of this bismuth component in the spectrum is in the order of 9% of the Bi spectral weight. The binding energy of the shoulder shifts towards metallic Bi^22^. Thus, we attribute this component to a small contribution of Bi atoms in a sub-stoichiometric environment such as e.g. in Bi_*x*_Te phase.

**Figure 6:**
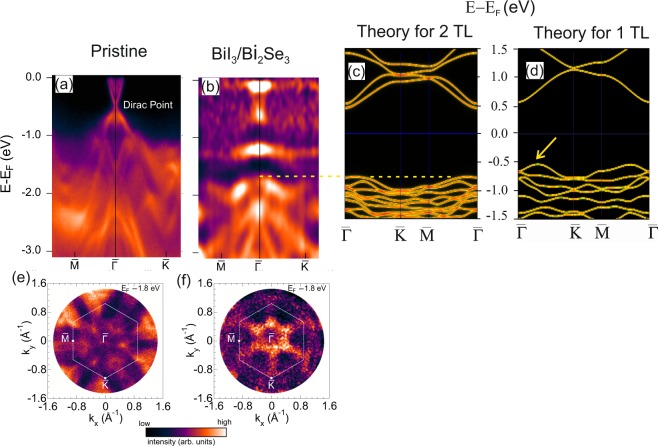
Band structure of pristine Bi_2_Se_3_(0001) (**a**) and BiI_3_ with saturation (**b**) coverage (about 1.5 monosheets) on Bi_2_Se_3_ measured along the $\bar{M}$-$\bar{{\rm{\Gamma }}}$-$\bar{K}$ direction. Spectra were collected at 130 K sample temperature at a photon energy of h*ν* = 40 eV and p-polarized light of the incoming beam. Calculated band structure for a free standing two (2TL) and single TL (1TL) thick film is shown in (**c**) and (**d**), respectively. The arrow emphasizes the difference in the band structure decisive for the presence of the indirect band gap. (**e**) Measured photoemission momentum map of pristine Bi_2_Se_3_(0001) and at full coverage of BiI_3_ on Bi_2_Se_3_ (**f**) at *E*_F_ = -1.8 eV. Note that, for a clear visualization of the band dispersion, the normalized intensity plot is presented in (**b**) and (**f**). The dashed white line in (**e**) and (**f**) indicates the substrate surface Brillouin zone.

Figures 6(a,b) compare the band dispersion along the $\bar{M}$-$\bar{{\rm{\Gamma }}}$-$\bar{K}$ high-symmetry direction of pristine and BiI_3_-saturated Bi_2_Se_3_ (0001), respectively. The latter corresponds to a complete BiI_3_ triple layer plus a second one which covers about a half of the surface area as found in the SXRD analysis. For pristine Bi_2_Se_3_(0001), the nearly linearly dispersive Dirac topological surface state (TSS) is clearly observed. Owing to the selenium vacancy induced n-doping, the Dirac point (D_*P*_) lies approximately 400 meV below the Fermi energy (E_*F*_).

Upon BiI_3_ adsorption, strong modifications of the band structure are observed. Fist of all, the TSS as such is destroyed and a new almost dispersion-less band appears at a binding energy (E_*B*_) of approximately −1.2 eV below E_*F*_. In the energy range between E = E_*F*_ and E = −0.7 eV two states at $\bar{{\rm{\Gamma }}}$ are observed which might be attributed to the conduction band (CB) of BiI_3_. For comparison Fig. 6(c) shows the calculated band structure for a 2TL thick BiI_3_ film including the atomic relaxations derived by the SXRD experiments. Our SXRD data suggest that the saturation film thickness is about one and a half TL’s. That is why we have chosen the calculation related to the two TL thick film for direct comparison with experiment.

The calculation for the free-standing 2TL film cannot reproduce all the interface- formation related new features, which are the new flat band interface states as well as the modification of the TSS. The energetic position of the calculated valence band (VB) maximum is aligned at $\bar{{\rm{\Gamma }}}$ so as to match the experiment in (b). This is emphasized by the dashed horizontal line. The VB is related to iodine states^5^ and is separated from the conduction band (CB) by a gap of approximately E_*g*_ = 1.2 eV, which can be considered to be direct owing to the flat band conditions in the vicinity of the $\bar{{\rm{\Gamma }}}$ point. The CB of BiI_3_ can also be identified in the momentum resolved photoemission experiment, which has a considerably smaller dispersion as the calculated one for the free-standing two TL thick film. We note that the calculated band structure for a single TL film [see Fig. 6(d)] -albeit similar to the 2TL band structure- exhibits some differences such as a smaller number of bands, a somewhat smaller band gap (E_*g*_ = 1.08 eV as compared to 1.18 eV for the 2TL film) and -most importantly- an indirect gap, in good agreement with the calculations in ref.^5^. In the experiment there is no evidence for an indirect gap mediated by a CB state between the $\bar{{\rm{\Gamma }}}$ and the $\bar{K}$ point [see arrow in Fig. 6(d)], thus the experiment is in closer correspondence with the 2TL calculation as expected.

The substantial change in electronic states is also found in the two-dimensional (2D) (*k*_*x*_, *k*_*y*_) photoelectron momentum map. As an example we have chosen the energy E = −1.8 eV below E_*F*_. Figure 6(e,f) compare the 2D momentum map of the pristine Bi_2_Se_3_(0001) and the BiI_3_ covered Bi_2_Se_3_(0001) surface. Upon film deposition the photoemission intensity is greatly suppressed near surface BZ boundary but a new six-fold star shaped VB state appears centered at the $\bar{{\rm{\Gamma }}}$-point which is related to the iodine-derived VB. The sixfold instead of threefold symmetry is due to the presence of two rotational domains.

Thus, the momentum resolved photoemission experiment indicates a considerable modification of the electronic structure of both, the Bi_2_Se_3_(0001) surface and of the BiI_3_-film. At first view this comes as a surprise given the rather weak structural modification of the Bi_2_Se_3_(0001) surface, but recent theoretical studies have predicted the appearance of interface states even in vdW epitaxial interfaces^23,24^.

## Conclusions

We have shown that a stabile BiI_3_ monosheet can be grown on the Bi_2_Se_3_(0001) surface. The geometric structure of the BiI_3_ film -although basically reminiscent of the bulk- is characterized by strong relaxations along the vertical direction. Similarly, the electronic structure of the film and the Bi_2_Se_3_ surface are significantly modified. The TSS of the pristine Bi_2_Se_3_(0001) surface is instable against BiI_3_ adsorption and a new non-dispersing interface state appears in the direct band gap (1.2 eV) of BiI_3_.

## Methods

### Sample preparation

The Bi_2_Se_3_ single crystal was cleaned by mild Ar^+^ ion sputtering followed by annealing up to about 500 °C leading to a clean surface and to a sharp contrasted (1 × 1) low energy electron diffraction (LEED) pattern^11,12^. Presynthesized BiTeI was loaded into a Knudsen cell and heated up to 380 °C. Single crystals of BiTeI were obtained by the chemical vapor transport (CVT) technique starting from a stoichiometric mixture of Bi, BiI_3_ and Te. A sealed quartz ampoule with the reactants was placed into a two-zone furnace with a temperature gradient for 5 days. The reactants were held at 500 °C and the hexagonal platelets of BiTeI up to 5 mm in diameter have grown (Fig. 1a), by autotransport, directly on the batch. We used the optimum temperature gradient 500/450 °C reported for the gas-phase transport of BiTeI on the basis of comprehensive phase-diagram studies^7,8^. The chemical purity of the product was confirmed by X-ray energy dispersive analysis and powder X-ray diffraction that yielded, respectively, a slightly Te-deficient composition and a strong preferred orientation of the powdered sample in consistence with its high crystallinity.

### Surface X-ray diffraction

Surface X-ray diffraction experiments were carried out at the beamline BM25b of the European Synchrotron Radiation Facility in Grenoble (France) using a six-circle UHV diffractometer. Samples were prepared *in-situ* in the same way as in the LEED, STM, XPS and photoemission experiments. In total the integrated intensities of 2786 reflections were collected by performing scans along the reciprocal q_*z*_ = *l* × c* direction in reciprocal space and collecting the reflected beam by using a two-dimensional pixel detector. The I_*obs*_(hkl) were then multiplied by instrumental correction factors C_*corr*_ as outlined in detail in refs.^25,26^ yielding the experimental structure intensities [|F_*obs*_(hkl)|^2^] via: |F_*obs*_(hkl)|^2^ = I_*obs*_(hkl) × C_*corr*_. Subsequently, the |F_*obs*_(hkl)|^2^ were averaged according to the *p*3 plane group symmetry of the (0001) surface leading to in total 1042 symmetry independent |F_*obs*_(hkl)|^2^. The total uncertainty (1 *σ*) is estimated by the quadrature sum of the statistical and the systematic error as outlined e.g. in ref.^27^. We find the reproducibility of symmetry equivalent |F_*obs*_(hkl)|^2^ is equal to 17%.

### Momentum resolved photoemission

Momentum-resolved photoemission spectroscopy experiments were carried out to study the electronic states upon BiI_3_ deposition on pristine Bi_2_Se_3_(0001) surface. Here, p-polarized light with a photon energy of *hν* = 40 eV was used. The incident photon beam lies in the *k*_*y*_–*k*_*z*_ plane at an angle of 25° above the surface plane. By utilizing momentum microscopy, the wide acceptance angle of photoelectrons of ±90° can be simultaneously collected over a hemispherical region in real space. This provides full access to directly probe two-dimensional (*k*_*x*_, *k*_*y*_) reciprocal-space momentum maps of the photoemission intensity throughout the entire surface Brillouin zone (SBZ). Furthermore, by scanning the binding energy *E*_*B*_, the complete information of three-dimensional *E*_*B*_(*k*_*x*_, *k*_*y*_) maps, containing band dispersion along all directions of the SBZ, can be obtained^28^.

### First-principles calculations

First-principles simulations have been carried out using a full-potential full-relativistic Green function method within the multiple scattering theory, specially designed as for bulks and for semiinfinite systems such as surfaces and interfaces^29,30^. The calculations were performed within the density functional theory in a generalized gradient approximation^31^. The angular momentum cutoff was taken to be *l*_*max*_ = 3 and a Γ-centered (30 × 30 × 12) *k*-mesh was used.

## References

[1] Lehner AJ (2015). Electronic structure and photovoltaic application of bil_3_. Applied Physics Letters.

[2] Brandt, R. E. *et al*. Investigation of bismuth triiodide (BiI_3_) for photovoltaic applications. *The Journal of Physical Chemistry Letters* **6**, 4297–4302 PMID: 26538045 (2015). 10.1021/acs.jpclett.5b02022 26538045

[3] Ruck M (1995). Darstellung und Kristallstruktur von fehlordnungsfreiem Bismuttriiodid. Z. Kristallogr..

[4] Zhang W-B, Xiang L-J, Li H-B (2016). Theoretical perspective of energy harvesting properties of atomically thin bii3. J. Mater. Chem. A.

[5] Ma F (2015). Single layer bismuth iodide: Computational exploration of structural, electrical, mechanical and optical properties. Scientific Reports.

[6] Tusche C, Krasyuk A, Kirschner J (2015). Spin resolved bandstructure imaging with a high resolution momentum microscope. Ultramicroscopy.

[7] Oppermann H, Petasch U, Schmidt P, Keller E, Krämer V (2004). On the pseudobinary systems Bi_2_Ch_3_/BiX_3_ and the ternary phases in these systems (Ch=S, Se, Te; X=Cl, Br, I). ii: Bismuthselenidehalides Bi_2_Se_3_/BiX_3_ and bismuthtelluridehalides bi_2_te_3_/bix_3_. Z. Naturforsch..

[8] Oppermann H, Schmidt M, Schmidt P (2005). Autotransport oder selbsttransport systeme mit gasphasentransporten unter dem eigenen zersetzungsdruck. Z. Anorg. Allg. Chem..

[9] Binnewies, M., Glaum, R., Schmidt, M. & Schmidt, P. *Chemical Vapor Transport Reactions* (De Gruyter, Berlin, 2012).

[10] Zeugner A (2017). Modular design with 2d topological-insulator building blocks: Optimized synthesis and crystal growth and crystal and electronic structures of bi_*x*_tei (*x* = 2; 3). Chem. Mater..

[11] Roy S (2014). Tuning the dirac point position in bi2se3(0001) via surface carbon doping. Phys. Rev. Lett..

[12] Roy S (2014). Atomic relaxations at the (0001) surface of bi2se3 single crystals and ultrathin films. Phys. Rev. B.

[13] Gardes B, Brun G, Tedenac J (1989). Contribution to the study of the bismuth–selenium system. Eur. J. Solid State Inorg. Chem..

[14] Trotter J, Zobel T (1966). The crystal structure of SbI_3_ and BiI_3_. Zeitschrift f. Kristallographie.

[15] Devidas, T. R. *et al*. Pressure-induced structural changes and insulator-metal transition in layered bismuth triiodide, BiI_3_: a combined experimental and theoretical study. *Journal of Physics: Condensed Matter* **26**, 275502 http://stacks.iop.org/0953-8984/26/i=27/a=275502 (2014). 10.1088/0953-8984/26/27/275502 24934819

[16] Zucker UH, Perenthaler E, Kuhs WF, Bachmann R, Schulz H (1983). PROMETHEUS. a program system for investigation of anharmonic thermal vibrations in crystals. Journal of Applied Crystallography.

[17] Kuhs WF (1992). Generalized atomic displacements in crystallographic structure analysis. Acta Cryst. A.

[18] Shannon RD, Prewitt CT (1969). Effective ionic radii in oxides and fluorides. Acta Crystallographica Section B.

[19] Shannon RD (1976). Revised effective ionic radii and systematic studies of interatomic distances in halides and chalcogenides. Acta Crystallographica Section A.

[20] Schneider C (2012). Expanding the view into complex material systems: From micro-arpes to nanoscale haxpes. Journal of Electron Spectroscopy and Related Phenomena.

[21] Yeh JJ, Lindau I (1985). Atomic subshell photoionization cross sections and asymmetry parameters: 1 ≤ Z ≤ 103. Atomic Data and Nuclear Data Tables.

[22] Gibson, Q. D. *et al*. Termination-dependent topological surface states of the natural superlattice phase Bi_4_Se_3_. *Phys. Rev. B* **88** (2013).

[23] Eremeev SV, Tsirkin SS, Nechaev IA, Echenique PM, Chulkov EV (2015). New generation of two-dimensional spintronic systems realized by coupling of rashba and dirac fermions. Science Reports.

[24] Eremeev, S., Menshov, V., Tugushev, V. & Chulkov, E. Interface induced states at the boundary between a 3D topological insulator Bi_2_Se_3_ and a ferromagnetic insulator EuS. *Journal of Magnetism and Magnetic Materials* **383**, 30–33 http://www.sciencedirect.com/science/article/pii/S0304885314008518. Selected papers from the sixth Moscow International Symposium on Magnetism (MISM-2014) (2015).

[25] Vlieg E (1997). Integrated Intensities Using a Six-Circle Surface X-ray Diffractometer. J. Appl. Crystallogr..

[26] Schlepütz CM (2005). Improved data acquisition in grazing-incidence X-ray scattering experiments using a pixel detector. Acta Cryst. Section A.

[27] Robinson IK, Tweet DJ (1992). Surface X-ray diffraction. Reports Prog. Phys..

[28] Suga S, Tusche C (2015). Photoelectron spectroscopy in a wide hn region from 6 ev to 8 kev with full momentum and spin resolution. Journal of Electron Spectroscopy and Related Phenomena.

[29] Lüders M, Ernst A, Temmerman WM, Szotek Z, Durham PJ (2001). Ab initio angle-resolved photoemission in multiple-scattering formulation. Journal of Physics: Condensed Matter.

[30] Geilhufe M (2015). Numerical solution of the relativistic single-site scattering problem for the coulomb and the Mathieu potential. Journal of Physics: Condensed Matter.

[31] Perdew JP, Burke K, Ernzerhof M (1996). Generalized gradient approximation made simple. Phys. Rev. Lett..

